# Psychometric properties of the Hungarian Visceral Sensitivity Index (VSI-H): insights from two cross-sectional studies on self-reported IBS and gluten-related conditions

**DOI:** 10.1186/s40359-025-02918-3

**Published:** 2025-07-01

**Authors:** Eszter Bertalan, Zsolt Horváth, Panna Gajdos, Tímea Magyaródi, Adrien Rigó

**Affiliations:** 1 Institute of Psychology, ELTE Eötvös Loránd University, Izabella U. 46, Budapest, 1064 Hungary; 2 Doctoral School of Psychology, ELTE Eötvös Loránd University, Budapest, Hungary; 3 Centre of Excellence in Responsible Gaming, University of Gibraltar, Gibraltar, Gibraltar

**Keywords:** Irritable bowel syndrome (IBS), Gluten-related disorders, Anxiety, Gastrointestinal, Quality of life, Visceral Sensitivity Index (VSI)

## Abstract

**Background:**

The present study examines the role of gastrointestinal symptom-specific anxiety in gluten-related conditions (e.g., celiac disease, non-coeliac gluten sensitivity) and in irritable bowel syndrome (IBS). The Visceral Sensitivity Index is widely used instrument for assessing gastrointestinal symptom-specific anxiety, originally used among IBS individuals, but it proved applicable to other health conditions characterized with gastrointestinal symptom presence (e.g., inflammatory bowel disease, eating disorders). The coexistence and symptom overlap between IBS and gluten-related conditions may provide a rationale for investigating gastrointestinal symptom-specific anxiety in the latter population.

**Methods:**

Two cross-sectional studies were conducted; consisting of 308 self-reported IBS individuals (M_age_ = 36.46; SD_age_ = 11.08) and 341 individuals with self-reported gluten-related disorders (M_age_ = 34.48; SD_age_ = 12.18). Self-reported questionnaire assessed the level of gastrointestinal symptom-specific anxiety, trait anxiety, negative affectivity, perceived gastrointestinal symptoms in gluten-related conditions, quality of life and well-being.

**Results:**

The confirmatory factor analyses supported a unidimensional structure of the Hungarian version of the Visceral Sensitivity Index with adequate fit and high internal consistency in both samples. Invariance testing revealed variations in item interpretation, suggesting caution in statistical comparisons of Visceral Sensitivity Index scores. In the IBS sample, gastrointestinal symptom-specific anxiety showed medium-strong correlations with trait anxiety and IBS-specific quality of life, and among individuals with gluten-related conditions, it correlated with negative affectivity, gastrointestinal symptom frequency, and well-being. Among IBS subtypes, individuals with diarrhoea-predominant (IBS-D) and mixed-bowel habit (IBS-M) subtypes showed significantly higher Visceral Sensitivity Index scores compared to those with the unclassified subtype (IBS-U).

**Conclusions:**

The Hungarian Visceral Sensitivity Index proves reliable and valid for measuring gastrointestinal symptom-specific anxiety in IBS and gluten-related disorders. Thus, its relevance may extend to other conditions with prevalent GI symptoms, potentially linking both the physical and mental aspects of well-being and quality of life in these conditions.

**Supplementary Information:**

The online version contains supplementary material available at 10.1186/s40359-025-02918-3.

## Background

### GI symptom-specific anxiety

Frequently occurring abdominal pain and altered bowel function are hallmarks of chronic gastrointestinal (GI) conditions and often go together with gastrointestinal symptom-specific anxiety (GSA) [1, 2]. GSA, primarily in the context of irritable bowel syndrome (IBS), serves as an endogenous stressor directly influencing the perception and perpetuation of GI-specific symptoms. It may be associated with increased autonomic nervous system and neuroendocrine activity, heightened visceral sensitivity, and may contribute to symptom exacerbation and pain modulation. GSA manifests in three dimensions: cognitive, affective, and behavioral [3, 4]. Worry (catastrophizing thoughts), fear (learned response to harmless bodily sensations), vigilance (increased attention to GI sensations), sensitivity (increased perception of GI symptoms in certain situations), and avoidance (safety behaviors) comprise the dimensions of the behavioral and cognitive manifestations of GSA. Both interoceptive and contextual factors (such as proximity to lavatory facilities and eating environments) contribute to elevated GSA levels [3, 4].

### GSA in different health conditions

In disorders of gut-brain interaction (DGBI), where heightened distress sensitivity and impaired bidirectional brain-gut axis functioning prevail, GSA plays a central role [5, 6]. In these conditions, elevated generalized anxiety traits (e.g., neuroticism, trait anxiety) exacerbate catastrophizing tendencies, vigilance, and GSA, further reinforcing negative condition appraisals [1, 3]. IBS, the most recognized entity of DGBIs, affects 5–10% of the global population and has been thoroughly studied for its association with GSA [7–9]. According to the ROME IV criteria [10], IBS is classified into four subtypes, constipation-predominant (IBS-C), diarrhoea-predominant (IBS-D), mixed (IBS-M), and unclassified (IBS-U), based on patients' impaired bowel habit characteristics. Even though the pathomechanism of IBS remains unclear, most studies agree that it has a multifactorial origin [5, 8], involving genetic, immune, environmental, and psychosocial factors [8, 11]. Among psychological factors, GSA is of particular interest, as it can play a significant role in the pathomechanism, predicting IBS status, GI symptom severity [4, 12–15] and QOL [16–18]. Reducing GSA is usually considered an indicator in psychological IBS treatments, with positive changes suggesting improvement in IBS status post-intervention [1, 13, 18–20].

GSA has been notably linked to other conditions where GI symptoms prevail, including self-reported food hypersensitivity [21], eating disorders (EDs) [22, 23], and inflammatory bowel disease (IBD) [24, 25]. In this context gluten-related disorders (GRDs) may also merit attention. GRDs are now recognized to be widespread, with an estimated worldwide prevalence of 5%. The differential diagnosis remains obscure due to the broad spectrum of symptoms and the diverse etiology [26]. Coeliac disease (CD), wheat allergy (WA), gluten ataxia, dermatitis herpetiformis, and non-coeliac gluten/wheat sensitivity (NCGS/NCWS) are the most prominent elements of GRDs [26, 27]. In GRDs, gluten consumption is often linked to GI and extraintestinal symptoms, along with functional GI symptoms [28–30]. There is a notable comorbidity rate between GRDs (especially NCGS/NCWS) and IBS [29, 31]. Sainsbury et al. [32] and Parker et al. [30] observed that chronic DGBI symptoms are prevalent in CD patients, persisting despite strict adherence to gluten-free dieting and correlating with elevated psychological distress, somatization, reduced QOL [30], and elevated levels of GSA [33].

### Measuring GSA

The standard measure of GSA is the self-report Visceral Sensitivity Index (VSI) created by Labus and colleagues [3]. The questionnaire is widely used internationally and is available in various languages, including English [3, 4, 34], Japanese [14], Norwegian [21], Italian [25], and Ukrainian [35]. The VSI exhibits convincing psychometric properties and unidimensional factor structure [3, 4]. The VSI demonstrated excellent internal reliability coefficients (0.84–0.95) [3, 4, 14, 23–25, 34, 35] and is considered a valid instrument for measuring GSA. Positive correlations have been found between GSA and the severity and frequency of GI symptoms, pain catastrophizing, and distress, while negative associations with quality of life (QOL) [4, 12, 14, 15, 19]. Adaptation studies also underlined the VSI’s independent predictive value for IBS diagnostic status [4, 23], as well as its crucial mediating role in the complex relationship between general anxiety, anxiety sensitivity, and the severity of GI symptoms [4, 12, 13].

The VSI was originally developed to quantify GSA in IBS patients, although it has demonstrated reliable measurement properties in non-IBS individuals with predominant GI complaints, including Crohn’s disease, ulcerative colitis [24, 36], reflux hypersensitivity (RH), non-erosive reflux disease (NERD) [2], self-reported food hypersensitivity [21], EDs [22, 23], and other DGBI conditions [1]. Recent applications in IBD [24] and ED populations [23] have shown similar psychometric properties, though some items’ factor loadings differ, indicating the differential role of specific items in determining GI distress in such populations [23, 24]. However, to our knowledge, the GSA construct has not been thoroughly studied in GRD samples, nor has the VSI been adapted for its measurement.

### Aims

The current study aims to adapt the VSI into Hungarian and to assess its psychometric properties concurrently in IBS and GRD samples. For this purpose, internal reliability and factor structure of the VSI are examined and compared between the two samples. Additionally, to investigate its construct validity, the study analyzes the association of GSA with trait anxiety, IBS-specific QOL, and subtypes in the IBS group, alongside its relationship with negative affectivity (NA), perceived GI symptom frequency, and well-being in individuals with GRDs. Thus, the present study contributes to the adaptation of the VSI into a new language while also providing insights into its applicability across conditions characterized by prominent GI symptoms.

## Methods

### Procedure

To address the aims of the study, two distinct cross-sectional studies (Studies 1 and 2) were conducted among individuals with IBS and GRDs, respectively. Both studies used convenience sampling by means of an online survey, targeting adult individuals (≥ 18 years of age) from Hungary. The recruitment of the participants occurred online by sharing the questionnaire in thematic groups and forums focused on IBS patients and gluten-free dieting in Studies 1 and 2, respectively. In all cases, completion of the survey was preceded by acceptance of the informed consent form. The research and ethical protocols were approved by the relevant institutional review boards.

### Participants

To be included in Study 1, participants had to meet at least one of the following IBS-related eligibility criteria: 1. a self-reported medical diagnosis of IBS, or 2. a diagnosis of IBS based on ROME III IBS criteria, or 3. a diagnosis of IBS based on ROME IV IBS criteria. Despite the evident specificity of ROME IV [37–39], clinical practice shows that solely satisfying the stricter standards required by ROME IV may exclude patients who would benefit from IBS treatment [38]. This underscores the rationale for integrating ROME III and ROME IV criteria for participant inclusion. Both sets of criteria (see further: Measurements) might be considered to capture the spectrum-like nature of the condition.

In Study 2, three criteria were required to meet for the GRD sample inclusion: 1. self-reported GRD, including at least one of the following conditions (even based on medical diagnosis or self-declaration): coeliac disease (CD), non-celiac gluten/wheat sensitivity (NCGS/NCWS), wheat allergy (WA), gluten ataxia, or dermatitis herpetiformis; 2. adherence to a gluten-free diet; and 3. absence of a self-reported IBS diagnosis.

The presence of a mental disorder was not an exclusion criterion in either sample. While Study 1 included a question about self-reported mental disorders, Study 2 did not, as it aimed to recruit a general community sample of individuals with GRDs and/or following gluten-free dieting. Altered gut-brain axis often leads to the coexistence of IBS with anxiety and depression [40–42]. Consequently, participants with self-reported mental disorders (primarily anxiety and depressive disorders in Study 1) were not excluded, ensuring that a broader spectrum of mental health was represented in the IBS sample. Overall, both samples were consistent in including individuals with potential mental disorders.

The total cleaned sample comprised 367 and 815 participants in Studies 1 and 2, respectively. As a result of the inclusion and exclusion criteria, 308 and 341 individuals were included in the final IBS and GRD samples, respectively*. *Table 1 details the characteristics of the samples.

**Table 1:** Characteristics of the IBS and GRD samples

**Study 1 (IBS sample)** Total sample size: *N* = 367; Final sample size: *N* = 308 (100%)	
** Demographic characteristics in the final sample**	
Female gender	*N* = 274 (89.0%)
Mean age in years (SD)	36.46 (11.08)
** Clinical characteristics of the final samples**	
IBS medical diagnosis of IBS (self-reported)	*N* = 193 (62.7%)
Fulfilling ROME III	*N* = 245 (79.5%)
Fulfilling ROME III and IV	*N* = 199 (64.6%)
* Combination of IBS criteria:*	
Having only IBS medical diagnosis (self-reported)	*N* = 63 (20.5%)
Fulfilling only ROME III	*N* = 22 (7.1%)
Fulfilling only ROME III and IV	*N* = 93 (41.7%)
Having an IBS medical diagnosis (self-reported) and fulfilling ROME III	*N* = 24 (7.8%)
Fulfilling all criteria (ROME IV and IBS medical diagnosis)	*N* = 106 (34.4%)
* IBS subtypes:*	
IBS-C: constipation-predominant subtype	*N* = 51 (16.6%)
IBS-D: diarrhoea-predominant subtype	*N* = 138 (44.8%)
IBS-M: mixed-bowel habits subtype	*N* = 89 (28.9%)
IBS-U: unclassified subtype	*N* = 30 (9.7%)
Mean time since diagnosis in years (SD)	6.81 (5.64)
Self-reported food allergy and/or intolerance except GRDs	*N* = 175 (56.8%)
Self-reported psychiatric disorder	*N* = 65 (21.1%)
**Study 2 (GRD sample)** Total sample size: N = 815; Final sample size: N = 341 (100%)	
** Demographic characteristics in the final sample**	
Female gender	*N* = 322 (94.4%)
Mean age in years (SD)	34.48 (12.18)
** Clinical characteristics of the final samples**	
Medical diagnosis of GRD (self-reported)	*N* = 272 (79.77%)
* Presence of GRD condition:*	
Coeliac disease	*N* = 223 (65.4%)
NCGS/NCWS	*N* = 105 (30.8%)
Wheat allergy	*N* = 24 (7.0%)
Gluten ataxia	*N* = 5 (1.5%)
Dermatitis herpetiformis	*N* = 10 (2.9%)
* Frequency of GI symptom experienced after gluten consumption:*	
Gas/bloating	*N* = 259 (75.0%)
Abdominal pain	*N* = 208 (61.0%)
Epigastric pain	*N* = 179 (52.5%)
Diarrhoea/steatorrhoea	*N* = 179 (52.2%)
Nausea/vomiting	*N* = 93 (27.3%)
Constipation	*N* = 78 (22.9%)
Gastrointestinal reflux	*N* = 75 (22.0%)
Loss of appetite or weight loss	*N* = 43 (12.6%)
Haven’t experienced any symptoms	*N* = 34 (10.0%)

### Measurements

The *Visceral Sensitivity Index* [3] measures GI symptom-specific anxiety, originally developed for the IBS population. It consist of 15 unidirectional items that refer to the cognitive (e.g., evaluation of unpleasant GI sensations) and behavioral (e.g., avoiding risky situations) aspects of GSA (e.g., „I often worry about problems in my belly”). Participants rated their agreement on a 6-point Likert scale (0 = Strongly disagree; 5 = Strongly agree). With a total score ranging from 0–75, a higher value potentially indicates an increased GSA level. Permission to translate and adapt the VSI into Hungarian was granted by the authors [3] involved in the development of the instrument. A standard translation method [43] was followed to develop the Hungarian version of the VSI (VSI-H). Three independent psychologists working in the field of clinical health psychology first translated the VSI into Hungarian. An independent bilingual person translated the Hungarian translation back into English, and then the two English versions were compared. Finally, a group of experts created the consensual version of the VSI-H. The VSI-H was administered on both samples.

### Study 1

The gold-standard self-report diagnostic criteria, the *ROME IV Diagnostic Questionnaire for Functional Gastrointestinal Disorders in Adults (R4DQ) IBS module* [37, 44] was used to determine IBS group membership and to classify IBS subtypes. The IBS module consists of five questions and an additional item to define the following subtypes of IBS: constipation-predominant (IBS-C); diarrhoea-predominant (IBS-D); mixed-bowel habits (IBS-M); unclassified IBS (IBS-U). The following criteria must be met in order to obtain a diagnosis of IBS: 1) recurrent abdominal pain 2) accompanied by at least two of the following conditions: related to defecation, accompanying changes in bowel movement frequency, or alterations in stool consistency 3) persisting for at least six months. For the last criterion, an exception was made for IBS group membership stating that symptoms were not required to be present for at least 6 months, so meeting the modified ROME III criteria was sufficient for group membership. Items were rated on different scales, primarily based on the frequency of the phenomenon occurring in the last period. The questionnaire was adapted into Hungarian in accordance with the guidelines of the Rome Foundation [45].

The *State-Trait Anxiety Inventory – Trait Anxiety* (STAI-T) [46, 47] subscale is a 20-item self-report questionnaire, which is scored on a 4-point Likert scale (1 = Not at all so; 4 = Very much so). A higher score implies greater trait anxiety.

The *IBS-36 Quality of Life Measure for IBS* [44, 48] comprises 36 items, which present an overview of the life domains affected by IBS. The questions are answered on a 7-point Likert scale (1 = Never; 7 = Always) for the past two months. The initial questionnaire scoring ranged from 0 to 216. However, the current study used reverse scoring and mean item scores, indicating that a higher value suggests a better quality of life in IBS.

### Study 2

Study 2 aimed to investigate the diversity of perceived GI symptoms associated with gluten intake in both the general and GRD populations, thus a symptom checklist was constructed in the present study based on GRD guidelines [28, 49]. As adherence to a gluten-free diet was an inclusion criterion for Sample 2, no time period was specified, as the symptoms might disappear due to gluten avoidance. Participants were asked to select from a list of GI symptoms (in Table 1) that they had experienced following gluten intake, or to declare if they had not detected any symptoms. The number of perceived GI symptoms related to gluten ingestion (GRD symptoms) was treated as an aggregated variable ranging from 0 to 8. Higher scores on this scale indicate increased GI symptom perception associated with gluten consumption.

The *Positive and Negative Affect Schedule (PANAS)* [50, 51] is a widely used assessment of positive (PA) and negative (NA) affect. The 20-item mood scale consists of 10 positive and 10 negative items, each scored on a 5-point Likert scale (1 = Very slightly or not at all; 5 = Extremely). The participants are asked to rate their general tendency (i.e., not in a state-like manner) to experience each of the emotional states. The present study solely used the NA scale, with higher scores indicating a higher NA trait.

The abbreviated 5-item Hungarian version of the *WHO Well-Being Questionnaire (WHO-5)* [52, 53] was applied for evaluating general well-being over the past two weeks. In the questionnaire, respondents are asked to rate statements on a 4-point Likert scale (0 = Not at all typical; 4 = Quite typical), such as “During the past two weeks, have you felt cheerful and in good spirits?”. Higher scores on the measure indicate higher psychological well-being.

## Statistical analyses

Data were analyzed using SPSS 29.0 and Mplus 8.0 [54] statistical software. First, CFA were performed on both samples to test the model fit of the original single-factor structure of the VSI [3, 4]. In this model all 15 items loaded on the latent factor of GSA. The 15 items of the VSI were treated as continuous observed variables. The maximum likelihood robust to non-normality (MLR) estimation method was used for all CFA analyses due to violations of the normal distribution assumption. As a first step, the single-factor model was tested separately on the samples of Studies 1 and 2 (i.e., IBS and GRD samples, respectively). To evaluate the model’s goodness of fit the following parameters were considered. For the comparative fit index (CFI) and the Tucker-Lewis index (TLI), values > 0.95 indicated optimal, > 0.90 indicated adequate, and < 0.90 indicated poor model fit. For the root-mean-square error of approximation (RMSEA) and the standardized root mean squared residual (SRMR) indicators, values < 0.05 indicated excellent, < 0.08 indicated adequate, > 0.08 indicated poor model fit. If at least adequate model fit was not clearly established by all parameters, the results of the modification indices were also taken into account by allowing the error covariance between indicators. The internal consistency of the instrument was considered acceptable with Cronbach’s alpha coefficients ≥ 0.8.

Then, three levels of measurement invariance related to the one-factor model of the VSI were tested to determine the extent of measurement equivalence between samples of Studies 1 and 2 (i.e., IBS and GRD samples, respectively). At the first level of invariance (configural), the factor loadings and intercepts were allowed to differ between the two samples. At the second (metric) level, the factor loadings constrained to be equal, while intercepts could vary between the two samples. In the most restrictive (scalar) invariance model, factor loadings and intercepts were constrained to be equal across between the groups. The invariance models were evaluated based on the goodness of fit indicators. Next, the level of invariance was assessed by comparing subsequent invariance models and examining fit deterioration against the more restrictive models. The level of more restrictive invariance was accepted if the fit deterioration did not exceed the thresholds of 0.010 in the case of CFI and SRMR, and 0.015 in RMSEA [55].

For examining the associations between the variables, Spearman correlations were applied, as the variables showed marginal deviations from the normal distribution. In the IBS sample (Study 1), correlations of GSA (measured by the VSI total score), IBS QOL (IBS-36 score), and trait anxiety (STAI-T score) were tested. IBS subtypes, determined by the ROME IV R4DQ IBS module, were compared in terms of GSA (VSI total score) using one-way analysis of variance (ANOVA) with Bonferroni-corrected post-hoc tests. To account for the potential confounding effect of psychiatric disorders [40, 42], VSI scores were compared between participants with and without self-reported psychiatric disorders using an independent samples t-test. For the GRD sample (Study 2), we also correlated GSA (VSI total score) with GRD GI symptoms, well-being (WHO-5 score), and negative affectivity trait (PANAS-NA score).

## Results

### Descriptives

The descriptive statistics from both studies are summarized in Table 1.

Internal consistency coefficients of the applied measures are reported in Table 3. All instruments demonstrated high internal consistencies in both samples.

### CFA

**Fig. 1:**
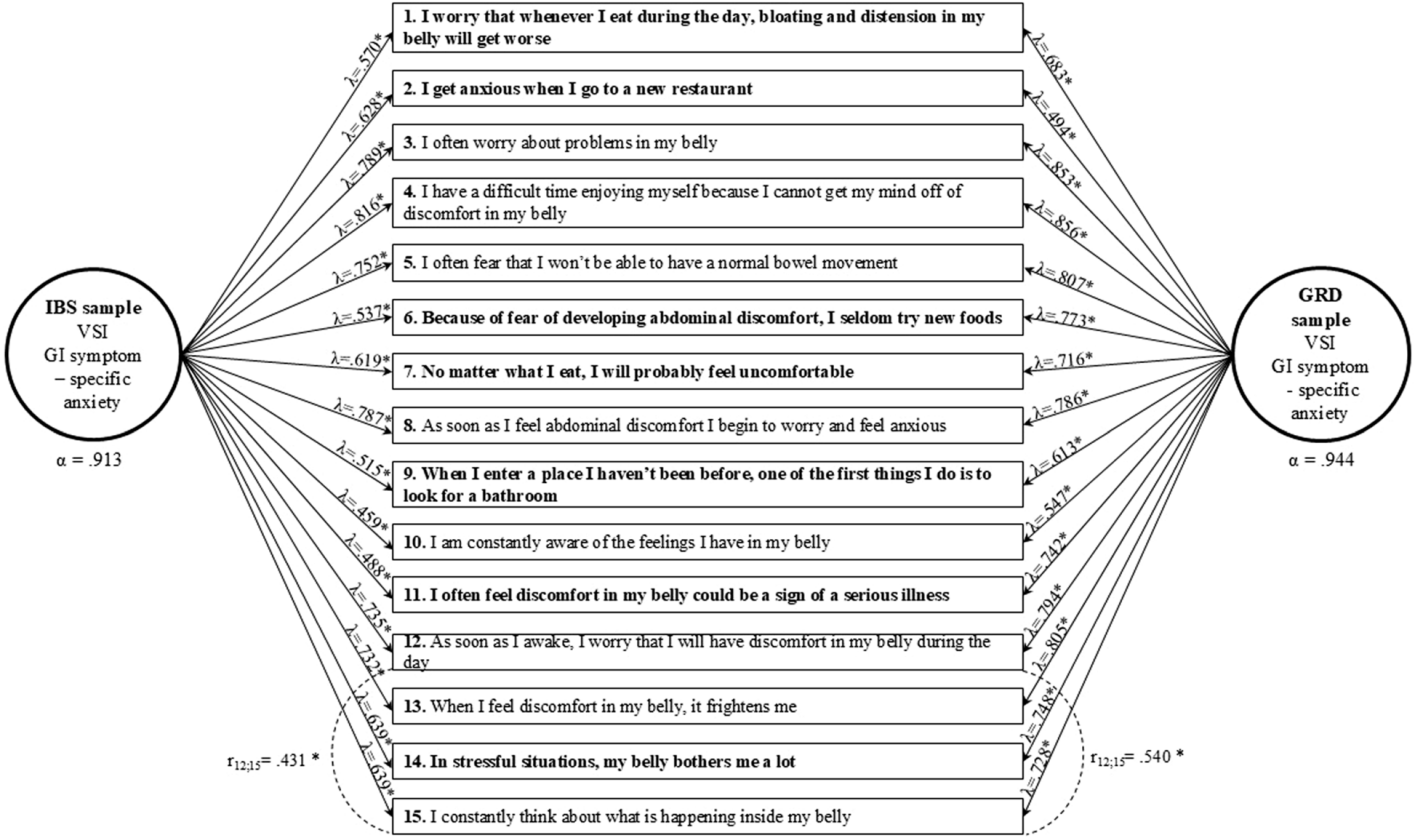
Standardized factor loadings and correlations of the final single-factor models of the Visceral Sensitivity Index (VSI) for Study 1 (IBS sample) and Study 2 (GRD sample)

The fit indices of the CFA analyses are presented in Table 2*.* In the IBS sample (Study 1), the fit of the first single-factor model (Model 1) was adequate based on the RMSEA, SRMR, and CFI, and close-to-adequate for the TLI. Similarly, the single-factor model (Model 1) in the GRD sample (Study 2) was slightly above the cutoff suggesting adequate fit based on the CFI and TLI, the RMSEA indicated close-to-adequate fit, while the SRMR showed an excellent model fit. Although adequate model fit was observed based on most indicators for Model 1, not all of them showed consistently at least an adequate fit in the samples, so modification indices were also examined (Table 2*, *Fig. 1). In both samples, the error covariance between items 12 (“As soon as I wake up, I worry that I will have discomfort in my stomach during the day”) and 15 (“I keep thinking about what is happening in my stomach”) was allowed due to possible overlap in their content and phrasing (Table 2*, *Fig. 1). The single-factor models that allowed for error covariation (Model 2) exhibited adequate or optimal model fit for all indices in each sample (Table 2*.* In the final models, all factor loadings were significant and moderate to strong across the samples, as shown in Fig. 1. Error correlations between items 12 and 15 were also significant and positive, in the IBS sample with moderate (Study 1) and in the GRD sample with strong correlations (Study 2). Finally, VSI exhibited high internal consistency in both samples (Fig. 1*; *Table 3)*.*

**Table 2:** Model fit and invariance testing of the VSI-15 models

	χ2	df	RMSEA (Cfit)	CFI	TLI	SRMR
**CFA measurement models – Study 1: IBS Sample (*N* = 277)**						
Model 1: Single-factor model	228.184***	90	0.074 (*p* = 0.001)	0.913	0.899	0.050
Model 2: Single-factor model, allowing for error covariance	186.000***	89	0.063 (*p* = 0.050)	0.939	0.928	0.047
**CFA measurement models – Study 2: GRD Sample (*N* = 262)**						
Model 1: Single-factor model	266.086***	90	0.086 (*p* < 0.001)	0.914	0.900	0.041
Model 2: Single-factor model, allowing for error covariance	204.985***	89	0.071 (*p* = 0.005)	0.943	0.933	0.038
**Measurement invariance testing (for Model 2)**						
Configural invariance model	391.698***	178	0.067 (*p* = 0.001)	0.942	0.931	0.043
Metric invariance model	463.751***	192	0.072 (*p* < 0.001)	0.926	0.919	0.073
Scalar invariance model	529.435***	206	0.076 (*p* < 0.001)	0.912	0.910	0.084
**Invariance model comparisons (for Model 2)**	**Δχ2**	**Δdf**	**ΔRMSEA**	**ΔCFI**	**ΔTLI**	**ΔSRMR**
Metric against the configural model	83.150***	14	−0.005	−0.016	−0.012	−0.030
Scalar against the metric model	68.876***	14	−0.004	−0.014	−0.009	−0.011

*IBS* irritable bowel syndrome, *GRD* gluten-related disorders

χ2: Chi-Square test statistics; RMSEA: Root Mean Squared Error of Approximation; Cfit: Closeness of fit test for RMSEA; CFI: Comparative Fit Index; TLI: Tucker-Lewis Index; SRMR: Standardized Root Mean Square Residual. Δχ2: Chi-square difference test. Significant Chi-Square tests or Chi-square difference tests are indicated by: ***p < 0.001. Non-significant: *p* > 0.05. In each difference in the invariance model test a value with a negative sign represents the decrease of the model fit

**Table 3:** Descriptive statistics of the validating variables and Spearman’s rank correlation matrix for construct validity in IBS and GRD samples (i.e., Study 1 and Study 2)

	Spearman’s correlations – in Study 1 – IBS sample (*N* = 244–308)				Spearman’s correlations – in Study 2 – GRD sample (*N* = 229–262)			
**Variables**	**1.VSI**	**2. STAI-T**	**3. IBS −36**	**Variables**	**1.VSI**	**5. PANAS-NA**	**6. GI symptom frequency in GRD**	**7. WHO-5**
**2.**	0.51***	-	-	**5.**	0.49**	-	-	-
**3.**	−0.58***	−0.38***	-	**6.**	0.40**	0.15*	-	-
	-	-	-	**7.**	−0.40**	−0.59**	−0.15*	-
**Mean**	46.91	53.53	2.12		27.87	2.17	3.27	12.61
**Min–Max**	8–74	23–77	0.00–5.25		0–74	– 1—4.8	0—7	5—20
**SD**	14.34	9.89	1.10		18.42	0.77	1.85	3.01
**Cronbach’s α**	0.91	0.91	0.94		0.94	0.88	-	0.83

*Abbreviations*: *IBS* irritable bowel syndrome, *GRD sample* gluten-related disorders, *VSI* Visceral Sensitivity Index measuring GI symptom-spec. anxiety, *STAI-T* Spielberger State-Trait Anxiety Inventory – Trait anxiety, *IBS-36* IBS Quality of Life Measure, *PANAS – NA *Positive and Negative Affect Schedule – Negative Affectivity, *WHO-5* The World Health Organisation- Five item Well-Being Index

*p < 0.05, **p < 0.01, ***p < 0.001

### Invariance testing

The results of the invariance testing are summarized in Table 2*.* The configural and metric models showed an adequate or optimal model fit. However, the CFI and SRMR showed a higher degree of fit deterioration than the given cut-offs, allowing only configural level invariance between the two samples to be confirmed. That is, the one-factor structure was comparable, but the factor loadings and thresholds were different between the two samples.

### Correlations

The findings of Spearman’s rank correlation analyses are presented in Table 3*.* In the IBS sample (Study 1), VSI showed a significant, positive and strong relationship with trait anxiety (STAI-T), and a significant, negative and strong correlation with IBS QOL (IBS-36). Within the GRD sample (Study 2), VSI demonstrated significant, moderate and positive correlations with NA (PANAS-NA) and GI symptoms and a negative, moderately strong and significant correlation with overall well-being (WHO-5).

### Between group comparisons

**Fig. 2:**
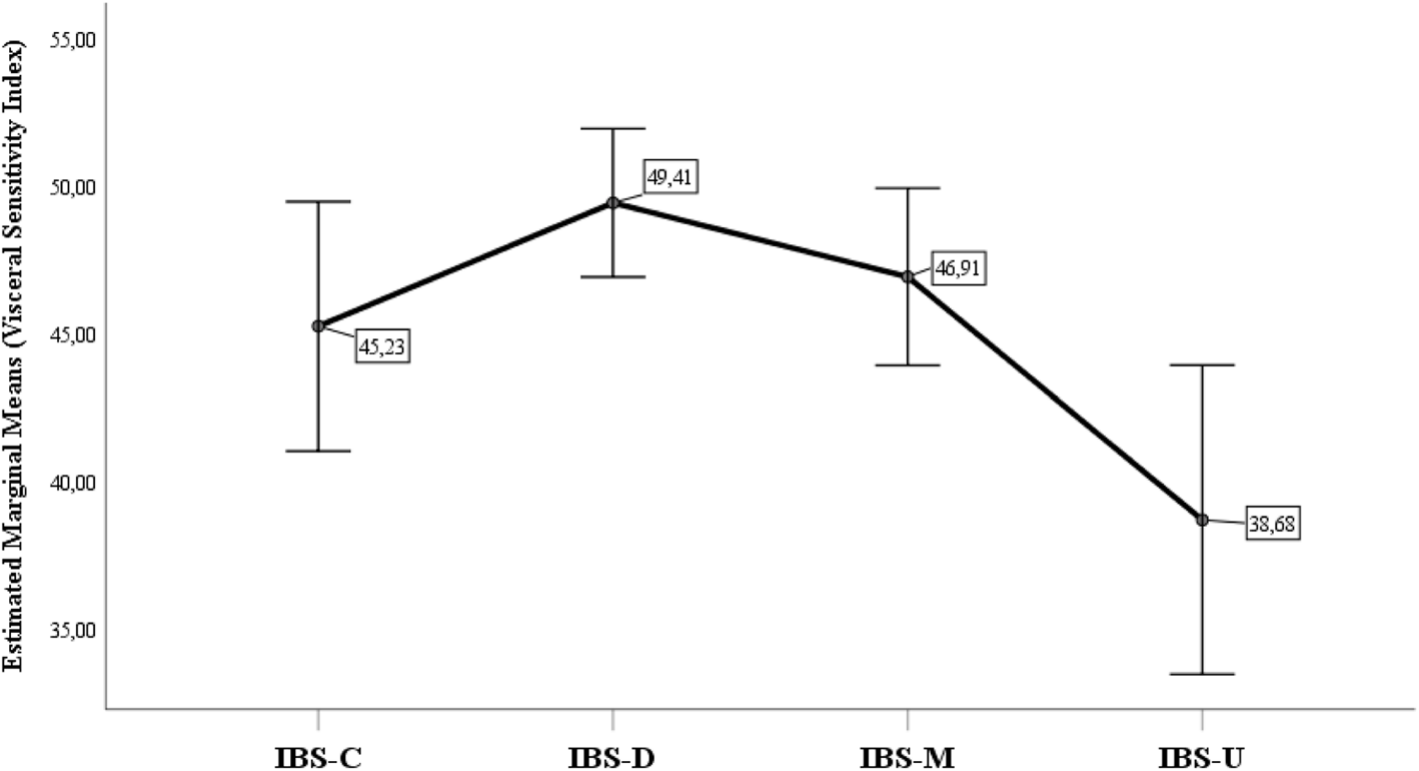
Comparison of Irritable Bowel Syndrome (IBS) subtypes based on Visceral Sensitivity Index (VSI) total scores. Notes: Group mean scores are shown as squares, with vertical lines representing 95% confidence intervals (95% CI). IBS-C: constipation-predominant; IBS-D: diarrhoea-predominant; IBS-M: mixed-bowel habits; IBS-U: unclassified subtype

The one-way ANOVA revealed a significant and moderate overall difference in VSI scores among IBS subtypes (F = 4.68; p = 0.003; η^2^ = 0.049). Figure 2 shows the mean VSI scores for each IBS subtype. Bonferroni-corrected post-hoc pairwise comparisons showed significantly higher VSI scores in individuals with IBS-D (p_Bonferroni_ = 0.002) and IBS-M (p_Bonferroni_ = 0.046) compared to those with the IBS-U subtype.

A non-significant and weak difference was observed in VSI mean scores between individuals with (M = 49.77, SD = 13.97) and without (M = 46.12, SD = 14.36) self-reported psychiatric disorders (t[275] = 1.75, *p* = 0.081, Cohen’s d = 0.26).

## Discussion

Based on our results, the Hungarian version of the Visceral Sensitivity Index (VSI) [3] showed a unidimensional factor structure for both self-reported IBS and GRD samples, with adequate fit indicators and high internal consistency. The invariance analyses do not support the statistical comparison of GSA (VSI) scores between GRD and IBS samples, suggesting that GSA reflects GI distress in both conditions, albeit with potential differences in item interpretation and relevance. The correlational analyses indicate that GSA is a related yet distinct construct from negative affectivity and trait anxiety and demonstrating medium to strong associations with symptom frequency and overall well-being in the GRD sample, as well as with IBS-specific QOL in the IBS sample. Additionally, differences in GSA among IBS subtypes were investigated, revealing that unclassified IBS (IBS-U) had significantly lower GSA than subtypes characterized by diarrhoea (IBS-D, IBS-M). Overall these findings support the construct validity and reliability of the Hungarian version of the VSI. Consequently, the VSI appears to be a promising instrument for assessing GSA in conditions characterized by heightened GI tract-related symptoms, whether of functional or organic origin.

To confirm the previously outlined unidimensional model of VSI [3, 4, 14, 23, 24, 35] and obtain fit indicators at an adequate level, an error correlation was allowed between items 12 and 15 for both samples in CFA. Our results are consistent with prior psychometric findings [3, 4, 14, 23, 35] including the previously noted higher inter-item correlation for item 12 [24]. Based on preliminary results, it can be expected that GSA levels would also be elevated in the non-IBS sample (e.g., self-reported food hypersensitivity, IBD, NERD, RH, functional heartburn, Crohn’s disease, CD) [2, 21, 24, 33, 36], but remain lower compared to the IBS sample [3, 24, 36]. A novel finding is that GSA is interpretable and measurable in both populations due to configural invariance, but metric and scalar disparities suggest that item assessment may differ, requiring caution in statistical comparison.

Differences in the interpretation of the items between the two groups can be understood from multiple perspectives. The relevance of GI distress may stem from the clinically significant interplay between the two conditions [28, 29, 31, 33]. While IBS and GRDs often exhibit high comorbidity [31] both can manifest in similar ways, including GI and extraintestinal symptoms, bidirectional brain-gut axis dysfunction, increased permeability, altered immunological functions, and high visceral sensitivity. However, the underlying cause attributed to their symptoms, as well as the patients' experiences, may differ [31]. For IBS, the condition's etiology is complex, and patients typically attribute their symptoms to the consumption of certain foods, which are often characterized by unpredictability and uncertainty, contributing to persistent vigilance, reduced perceived control and diminished QOL [56]. In gluten-related conditions, where the symptoms primarily attributed to gluten/wheat consumption, the strict lifestyle management, the diagnostic process, and the chronicity of the condition often serve as a source of anxiety and reduced QOL [57, 58]. Adherence to gluten-free diet frequently improves QOL, anxiety, and mood disorders in GRD individuals [33], but with comorbid IBS present, symptoms and QOL may not fully resolve despite strict dietary adherence [30, 31].

A second approach focuses directly on the VSI items, and how they reflect specific characteristics of the two conditions. When examining factor loadings of the VSI items (Fig. 1), the GRD sample exhibits higher factor loadings, especially for items reflecting new situational or activity-based determinants (e.g., “Because of fear of developing abdominal discomfort, I seldom try new foods”). This suggests that heightened GSA in GRD individuals may be linked to aspects of life management and unknown external experiences (e.g., unpleasant symptoms when visiting a new place or being more apprehensive about cross-contamination). Meanwhile, according to Black and colleagues' [34] VSI model, a heightened and persistent level of awareness and worry may play a more influential role in determining symptom severity in IBS rather than constant fear of consequences and relapse. Differences in item interpretation have also been reported in other studies, such as in the 13-item version of the VSI adaptation for ED population [23], where some items were more strongly associated with certain ED types (e.g., restroom-seeking with purging behavior; or avoidance of new foods in relation to avoidant/restrictive food intake disorder), while Trieschmann and colleagues [24] made similar observations in the IBD population.

Prior studies have consistently demonstrated that GSA, as assessed by the construct of the VSI and supported by findings on its convergent validity, is a distinct construct from anxiety sensitivity [4, 14, 23], neuroticism [1], general anxiety [3, 4, 12, 14, 24] and depression [2, 4, 35]. Our study aligns with these findings, revealing moderate to strong positive relationships between GSA and trait anxiety and NA, supporting their interrelated yet distinct nature. This distinction is further supported by the small, non-significant difference in GSA between individuals with and without self-reported mental disorders in the IBS sample, which suggests that mental disorders (i.e., diminished mental health) does not fully account for the observed variability in GSA.

Another important question arising in relation to construct validity is how GSA relates to QOL and well-being indicators. The results indicate that GSA is strongly linked to disease-specific QOL in the IBS sample, likely reflecting the impact of physical symptoms on life management and overall QOL. Likewise, in the GRD sample, GSA showed a moderate association with the general mental well-being indicator. These findings suggest that GSA may play a comparable role in both the mental and physical aspects of QOL. While there are contradictory results concerning the relationship between IBS and GSA in relation to mental [12] or physical [18] dimensions of QOL, a recent study indicates that elevated GSA correlates positively with the simultaneous occurrence of substantial psychological distress and pronounced GI symptoms, such as high symptom severity, abdominal pain, diarrhoea, and urgency [59]. For gluten-related conditions, Sainsbury and colleagues [60] found that psychological symptoms and maladaptive coping strategies had a greater impact on QOL in coeliac disease than GI symptoms.

Additionally, the construct validity of the Hungarian version of the VSI is further supported by the significantly lower GSA levels observed in individuals with the IBS-U subtype. This finding aligns with a previous meta-analysis indicating that this subtype is associated with lower rates of depression and anxiety compared to other IBS subtypes [61]. Furthermore, the absence of significant differences in VSI scores among IBS-C, IBS-D, and IBS-M subtypes in the present study is consistent with prior research [62]. Notably, individuals who experienced diarrhoea more frequently reported significantly higher GSA levels than those with IBS-U. This may be explained by their greater uncertainty, pronounced fear of incontinence, and reduced health-related QOL [63].

### Practical relevance and further directions

Increased GI distress can perpetuate the persistent fear of symptoms, especially with catastrophizing tendencies [1, 15]. Even with strict dietary adherence, it may contribute to maladaptive coping and unnecessary restrictive eating [22]. Therefore, reducing GSA has emerged as a key objective in psychological interventions for conditions with significant GI events affecting health status and QOL assessment [2, 12, 13, 16, 18, 64, 65]. Prior studies have proposed diverse psychological approaches for reducing GSA and IBS symptoms [20], including cognitive-behavioral therapy (CBT) [64], gut-directed hypnotherapy [65], and acceptance and commitment therapy [16, 18]. For GRDs, interventions may include elements of CBT and often target behavior change, primarily to increase dietary adherence [66], but they may also help reduce maladaptive beliefs and distress [33]. Further investigations could focus on how individuals' perception and acceptance of their health status influence the impact of GSA on the evaluation of GI symptoms and QOL [16, 19]. Additionally, exploring the interplay between psychological determinants, such as catastrophizing tendencies and coping styles, in GI-tract related conditions remains essential [19, 33]. Finally, future studies should aim to test the potential mediating role of GSA between psychological characteristics (e.g., trait NA and anxiety, anxiety sensitivity) and disease-specific outcomes (e.g., QOL, GI symptom severity) among individuals with IBS and GRDs [4, 12, 13].

## Limitations

Several limitations should be considered when interpreting the present findings. The cross-sectional design prevents the examination of causal relationships between variables. Convenience sampling resulted in non-representative IBS and GRD samples, characterized by an overrepresentation of women and resulting limited ethnic diversity. Furthermore, the online data collection may have introduced bias, as some participants might have been reluctant to disclose sensitive psychological or disorder-specific information. The absence of diagnostic evidence and reliance on self-report measurements, exacerbated by the restrictions during the COVID-19 pandemic, may led to diagnostic inaccuracies. Despite efforts to distinguish between IBS and GRD samples, overlaps could still occur. Furthermore, due to the clinical focus of Study 1, different measures were used across the two studies, limiting the direct comparability of associations. For instance, self-reported mental disorders were assessed only in the IBS sample, preventing the evaluation of their potential confounding effect in individuals with GRDs. Furthermore, the symptom frequency indicator used in the GRD sample was non-standardized and potentially less reflective of symptom severity, while no standardized measure was available in the IBS sample either. Moreover, we focused exclusively on GI symptoms, whereas extraintestinal symptoms, which are also typical in these conditions and impact QOL, were not considered [18, 34]. Finally, the factor analyses indicated relatively lower loadings (< 0.6) for some items, with a notable decrease in item 10 across both populations compared to prior analyses [3, 4, 14, 23]. The discrepancy may stem from the Hungarian phrasing of the item, warranting further modification.

## Conclusions

In conclusion, to our knowledge, this is the first study to thoroughly investigate the relevance of GSA and the applicability of the VSI in gluten-related conditions alongside IBS. Based on the findings of the invariance testing, one should carefully evaluate the VSI scores and critically consider the relevance of each GSA element, taking into account the clinical and psychological characteristics of the given population. For future studies, it could be essential to conduct additional clinical investigations across these conditions (e.g., DGBIs, GRDs, IBD and even EDs), ideally applying longitudinal designs with clearly defined clinical groups. Adopting a biopsychosocial approach could further deepen our understanding of how the interplay between psychosocial and physiological factors affects one's QOL. Finally, the present study contributed to the adaptation of the VSI into a new language, extending its applicability in international research.

## Supplementary Information

Supplementary Material 1.

## Data Availability

The datasets analyzed during the current study are available in the Open Science Framework (OSF) repository, https://osf.io/u9ycd/.

## Abbreviations

b: Unstandardized regression coefficient
β: Standardized regression coefficient
CBT: Cognitive-behavioral therapy
CD: Coeliac disease
CFA: Confirmatory factor analysis
CFI: Comparative fit index
CI: Confidence interval
DGBI: Disorder of gut-brain interaction
ED: Eating disorder
GI: Gastrointestinal
GRD: Gluten-related disorder
GSA: Gastrointestinal symptom-specific anxiety
IBD: Inflammatory bowel disease
IBS: Irritable bowel syndrome
IBS-C: Constipation-predominant subtype of irritable bowel syndrome
IBS-D: Diarrhoea-predominant subtype of irritable bowel syndrome
IBS-M: Mixed-bowel habits subtype of irritable bowel syndrome
IBS-U: Unclassified subtype of irritable bowel syndrome
MLR: Maximum likelihood robust to non-normality
NA: Negative affectivity
NCGS/NCWS: Non-coeliac gluten/wheat sensitivity
NERD: Non-erosive reflux disease
PA: Positive affect
PANAS: Positive and Negative Affect Schedule
QOL: Quality of life
R4DQ: ROME IV Diagnostic Questionnaire for Functional Gastrointestinal Disorders
RH: Reflux hypersensitivity
RMSEA: Root-mean-square error of approximation
SRMR: Standardized root mean squared residual
STAI-T: State-Trait Anxiety Inventory – Trait Anxiety
TLI: Tucker-Lewis index
VSI: Visceral Sensitivity Index
VSI-H: Hungarian version of the Visceral Sensitivity Index
WA: Wheat allergy
WHO-5: 5-Item WHO Well-Being Questionnaire
