# Supplementary material for: Psychometric properties of the Hungarian Visceral Sensitivity Index (VSI-H): insights from two cross-sectional studies on self-reported IBS and gluten-related conditions
Source: BMC Psychol. 2025 Jul 1;13:679. doi: 10.1186/s40359-025-02918-3 (PMC12220099; doi:10.1186/s40359-025-02918-3)
Supplement: Supplementary file 1 — Supplementary Material 1. [file 40359_2025_2918_MOESM1_ESM.docx]

**The Hungarian version of the Visceral Sensitivity Index (VSI-H)** [1]

**A Viscerális Szenzitivitás Index magyar változata (VSI-H)**

Az alábbiakban olyan **állításokat talál, amik arra vonatkoznak, hogy az emberek hogyan reagálnak a kellemetlen tünetekre, diszkomfort érzésre a hasukban, gyomor-bélrendszerükben.** Ezek a tünetek magukba foglalják az ott megjelenő fájdalmat, hasmenést, székrekedést, puffadást. Kérjük, hogy valamennyi tételt figyelmesen olvassa el, és jelölje, hogy **milyen mértékben ért vele egyet, mennyire jellemző Önre az adott állítás!**

|  | Egyáltalán nem értek egyet | Nem értek egyet | Inkább nem értek egyet | Inkább egyetértek | Egyetértek | Teljes mértékben egyetértek |
| --- | --- | --- | --- | --- | --- | --- |
| 1. Aggódom, hogy ha napközben eszem, a hasamban érzett puffadás és felfúvódás rosszabb lesz. | 0 | 1 | 2 | 3 | 4 | 5 |
| 2. Szorongás fog el, amikor új étterembe megyek. | 0 | 1 | 2 | 3 | 4 | 5 |
| 3.Gyakran aggódom a hasi problémáim miatt. | 0 | 1 | 2 | 3 | 4 | 5 |
| 4. Nagyon nehéz kikapcsolódnom, mert nem tudom elterelni a figyelmem a hasi kényelmetlenség érzésről / rossz érzésről. | 0 | 1 | 2 | 3 | 4 | 5 |
| 5. Gyakran félek attól, hogy sosem lesz normális a bélműködésem / székelésem. | 0 | 1 | 2 | 3 | 4 | 5 |
| 6. Ritkán próbálok ki új ételeket, mert tartok a hasi kényelmetlenség érzéstől. | 0 | 1 | 2 | 3 | 4 | 5 |
| 7. Mindegy mit eszem, valószínűleg kényelmetlenül fogom magam érezni tőle. | 0 | 1 | 2 | 3 | 4 | 5 |
| 8. Amint kényelmetlen érzéseket tapasztalok a hasamban, azonnal aggódni és szorongani kezdek. | 0 | 1 | 2 | 3 | 4 | 5 |
| 9. Amikor egy olyan helyre érkezem, ahol korábban sosem jártam, az egyik első dolog, amit megteszek, hogy megkeresem a mosdót. | 0 | 1 | 2 | 3 | 4 | 5 |
| 10. Folyamatosan tudatában vagyok a hasi érzéseimnek / annak, hogy mit érzek a hasamban. | 0 | 1 | 2 | 3 | 4 | 5 |
| 11. Gyakran úgy érzem, hogy a hasamban tapasztalt kényelmetlenség érzés egy komoly betegség jele lehet. | 0 | 1 | 2 | 3 | 4 | 5 |
| 12. Amint felébredek, azonnal aggódni kezdek, hogy a nap során kényelmetlenséget fogok érezni a hasamban. | 0 | 1 | 2 | 3 | 4 | 5 |
| 13. A hasi kényelmetlenségérzés megijeszt engem / ijesztő számomra. | 0 | 1 | 2 | 3 | 4 | 5 |
| 14. Stresszes helyzetekben hasi érzéseim kellemetlenek számomra. | 0 | 1 | 2 | 3 | 4 | 5 |
| 15. Folyamatosan azon gondolkozom, hogy éppen mi történik a hasamban. | 0 | 1 | 2 | 3 | 4 | 5 |

Reference for the original version:

1. Labus JS, Bolus R, Chang L, Wiklund I, Naesdal J, Mayer EA, et al. The Visceral Sensitivity Index: development and validation of a gastrointestinal symptom-specific anxiety scale: THE VISCERAL SENSITIVITY INDEX. Alimentary Pharmacology & Therapeutics. 2004 Jul;20(1):89–97.
